# Gait Alteration Due to Haemophilic Arthropathies in Patients with Moderate Haemophilia

**DOI:** 10.3390/ijerph19127527

**Published:** 2022-06-20

**Authors:** Alban Fouasson-Chailloux, Fabien Leboeuf, Yves Maugars, Marc Trossaert, Pierre Menu, François Rannou, Claire Vinatier, Jérome Guicheux, Raphael Gross, Marc Dauty

**Affiliations:** 1 Inserm, UMR 1229, RMeS, Regenerative Medicine and Skeleton, Nantes Université, ONIRIS, 44042 Nantes, France; yves.maugars@chu-nantes.fr (Y.M.); pierre.menu@chu-nantes.fr (P.M.); claire.vinatier@univ-nantes.fr (C.V.); jerome.guicheux@univ-nantes.fr (J.G.); marc.dauty@chu-nantes.fr (M.D.); 2 Service de Médecine Physique et Réadapatation Locomotrice et Respiratoire, CHU Nantes, Nantes Université, 44093 Nantes, France; 3 Service de Médecine du Sport, CHU Nantes, Nantes Université, 44093 Nantes, France; 4 Institut Régional de Médecine du Sport, 44093 Nantes, France; 5 Movement-Interactions-Performance (MIP), EA 4334, CHU Nantes, Nantes Université, 44000 Nantes, France; fabien.leboeuf@chu-nantes.fr (F.L.); raphael.gross@chu-nantes.fr (R.G.); 6 School of Health & Society, The University of Salford, Salford M6 6PU, UK; 7 Service de Rhumatologie, CHU Nantes, Nantes Université, 44000 Nantes, France; 8 Centre Régional de Traitement de l’Hémophilie, CHU Nantes, Nantes Université, 44000 Nantes, France; marc.trossaert@chu-nantes.fr; 9 Service de Rééducation et de Réadaptation de l’Appareil Locomoteur et des Pathologies du Rachis, Hôpitaux Universitaires-Paris Centre, Groupe Hospitalier Cochin, Assistance Publique—Hôpitaux de Paris, 75014 Paris, France; francois.rannou@aphp.fr; 10 UFR Odontologie, CHU Nantes, Nantes Université, 44042 Nantes, France; 11 CHU Nantes, PHU4 OTONN, 44093 Nantes, France

**Keywords:** haemophilia, arthropathy, 3D gait analysis, walking, Haemophilia Joint Health Score

## Abstract

Some patients with moderate haemophilia (PWMH) report joint damage potentially responsible for gait disorders. Three-dimensional gait analysis (3DGA) is a relevant tool for the identification of complex musculoskeletal impairment. We performed an evaluation with 3DGA of 24 PWMH aged 44.3 ± 16.1 according to their joint status [Haemophilia Joint Health Score (HJHS) < 10 or HJHS ≥ 10] and assessed the correlation with the radiological and clinical parameters. Sixteen had HJHS < 10 (group 1) and eight had HJHS ≥ 10 (group 2). They were compared to 30 healthy subjects of a normative dataset. Both knee and ankle gait variable scores were increased in group 2 compared to the controls (*p* = 0.02 and *p* = 0.04, respectively). The PWMH of group 2 had a significant increase in their stance phase, double support duration, and stride width compared to the controls and group 1 (*p* < 0.01). Very low correlations were found for the ankle gait variable score with the ankle Pettersson sub-score (r^2^ = 0.250; *p* = 0.004) and ankle HJHS sub-score (r^2^ = 0.150; *p* = 0.04). For the knee, very low correlation was also found between the knee gait variable score and its HJHS sub-score (r^2^ = 0.290; *p* < 0.0001). Patients with moderate haemophilia presented a gait alteration in the case of poor lower limb joint status.

## 1. Introduction

Haemophilia is a congenital disease responsible for a deficiency in clotting factor VIII (haemophilia A) or IX (haemophilia B). The disease is categorised into three sub-groups according to the level of clotting factor: severe (<1%), moderate (1–5%), or mild disease (5–40%) [1]. This clotting factor deficiency is responsible for repetitive intra-articular bleeding, which may induce haemophilic arthropathy, one of the most severe long-term complications of haemophilia [2]. Haemophilic arthropathy is characterised by joint contractures and joint tissue destruction, especially on the knees and ankles [2,3]. It is responsible for pain, functional disability, and a decrease in the quality of life [4]. Patients with moderate haemophilia (PWMH), who represent 15 to 20% of all patients with haemophilia, are supposed to have a lower risk of joint bleeding and less arthropathies [5,6,7,8,9]. However, some studies suggest that some PWMH have early onsets of joint bleeding and have several bleeds a year [10]. Indeed, from 25% to 77.2% of the PWMH reported joint damage and developed several lower-limb haemophilic arthropathies with potentially functional disorders [5,8,9,11,12,13].

Three-dimensional gait analysis (3DGA) is a relevant and useful tool for the detection of gait deviation and the subsequent identification of musculoskeletal impairment [14,15,16]. Recently, 3DGA in patients with severe haemophilia seems to be a promising tool to detect early walking modifications and to correlate joint damage to walking impairments [17,18]. Several studies have shown that 3DGA is a reproducible method to assess walking disorders [17,19,20]. It appears useful in the detection of early joint damage as well as in the long-term assessment of gait in this population [17]. In contrast, data from 3DGA in patients with moderate haemophilia are lacking [17,21].

The present study aimed to perform a systematic evaluation with 3DGA of the PWMH followed in a French regional centre for the treatment of haemophilia in order to provide kinematic and kinetic data according to their joint status. Then, we aimed to assess the potential correlations between the clinical and gait analysis data in these patients.

## 2. Materials and Methods

### 2.1. Participants

The PWMH were included from November 2019 to September 2020. All of the PWMH over 18 years, followed in the Regional Centre of Haemophilia Treatment of Nantes University Hospital (France) were contacted by phone. Inclusion criteria were having a moderate haemophilia (A or B) and being over 18 years. Criteria of exclusion were inability to walk, refusal to participate, having had a lower limb joint bleed in the past 6 months, and other diseases with an impact on walking (neurological and unstable cardiorespiratory diseases). Out of the 44 eligible PWMH, 12 could not be contacted by phone, five declined to participate in the study and three were excluded. Finally, 24 PWMH were included. The mean age was 44.3 ± 16.1 years, mean weight was 75.9 ± 15.0 kg, and mean height was 173.0 ± 6.6 cm. Sixteen had haemophilia A and nine had haemophilia B. The mean clotting factor level was 3.0 ± 1.0%. All of the patients had on-demand treatment. The PWMH were compared to a control group of 30 healthy men from our normative dataset (height: 1.67 ± 0.1 m, 69.8 ± 15.0 kg, and BMI 24.8 ± 4.2 kg·m^2^) constructed by our clinical gait service for the 3DGA routine.

The study protocol was ethically approved by the “Comité de Protection des Personnes” of Ile de France IV (2019/28) under ID-RCB: 2019-A00625-52 and declared on ClinicalTrials.gov under the identifier NCT04024176. All of the patients included gave their written consent to participate to the study.

### 2.2. Three-dimension Gait Analysis

Patients walked barefoot and unassisted along a 12-m walkway, at self-selected speed. First, all patients performed three to six gait trials to become familiar with the gait lab. Eight optoelectronic ViconMX-F40 cameras (Oxford Metrics, Yarnton, UK) recorded the trajectory of the reflective markers positioned on the subjects according to the conventional gait model 2.3 (CGM2) [22]. The gait cycles were detected from force-plates and refined from video recording. Marker trajectories were low-pass filtered according to the Woltring procedure [23]. The force-plate measurements were processed with a low-pass Butterworth filter (4th order, cut-off frequency of 6 Hz). The usual spatio-temporal parameters (i.e., walking velocity, cadence, step length, percentages of the stance phase and double support) were calculated for each gait cycle. The gait profile score (GPS) was computed to quantify the overall deviation of joint kinematics compared to the control group [24,25]. The GPS averages the root mean square difference of each joint angle (a.k.a gait variable scores (GVSs)) from their respective normative values. Due to the particular involvement of both the ankle and the knee in haemophilia, we specifically noted GVSs for the sagittal plane kinematics of the knee and ankle [25]. All of the processing including modelling operation and gait processing (that is normalisation of gait cycles) was carried-out with the Python open source library, pyCGM2 [22] hosting CGM2 operations.

### 2.3. Haemophilia Joint Health Score

The Haemophilia Joint Health Score (HJHS) is a clinical score used to assess joint impairment. Initially designed for children with haemophilia [26,27], HJHS (version 2.1) has also demonstrated high reliability in adult patients [28]. The HJHS has multiple items for the elbow, knee, and ankle joints, with a total score for each of three domains ranging from 0 to 40, associated with a global gait score ranging from 0 to 4. The total HJHS ranges from 0 to 124. A HJHS over 10 points is usually considered a poor joint status [11,29]. As we analysed walking, we excluded elbow sub-scores to have a “lower limb HJHS”. Hence, we subdivided our population into two sub-groups, PWMH with lower limb HJHS < 10 (group 1), and PWMH with lower limb HJHS ≥ 10 (group 2).

### 2.4. Haemophilic Arthropathy Evaluation

We chose to focus our evaluation on lower limb joints due to their main impact on walking. To determine joint status, radiographic evaluations of the knees and ankles were performed by two highly experienced specialists in physical medicine and rehabilitation (PMR) using the Pettersson radiological score [30]. The Pettersson score is based on eight specific radiographic criteria (0 to 13 points) and has been validated by the World Federation of Haemophilia to assess haemophilic arthropathy [31]. Knee and ankle sub-scores were calculated for each joint as mean values of the right and left side. In the case of joint arthroplasty, the joint was excluded from the score.

### 2.5. Statistical Analysis

Statistical analyses were performed in the R environment 2.13 (R Development Core Team, Vienna, Austria). Quantitative variables were reported by descriptive statistics. The normal distribution of the data was assessed using Shapiro–Wilk tests. Quantitative variable comparisons between group 1 and group 2 were performed with either the Student’s *T*-tests for independent variables or Mann–Whitney tests; comparison of the type of haemophilia (A or B) frequency was performed with Kruskall–Wallis tests. For the 3DGA variables, we used an ANOVA followed by a Bonferroni post hoc to analyse the relationship between scores in group 1, group 2, and the control group. The threshold was set to 0.05. In PWMH, the association between the GPS or GVS with clinical score (i.e., HJHS sub-scores, Pettersson knee and ankle sub-scores) was given by Spearman correlation (r^2^). The correlation coefficient was interpreted using the usual rules [32]: strong correlation (r^2^ > 0.9); high (0.7 < r^2^ < 0.9); moderate (0.5 < r^2^ < 0.7); low (0.3 < r^2^ < 0.5); very low (r^2^ < 0.3).

## 3. Results

### 3.1. Characteristics of the Study Participants

Nineteen PWMH had previously experienced lower limb joint bleedings (79.1%). Six patients had previous knee bleedings, eight patients had ankle bleedings, and five had both. The mean HJHS total score was 10.4 ± 14.0 with a mean knee sub-score of 3.0 ± 4.2 and mean ankle sub-score of 4.4 ± 6.7. The mean knee and ankle sub-scores of the Pettersson score were 2.0 ± 2.3 and 3.3 ± 3.8, respectively (Table 1). Sixteen patients had a lower limb HJHS < 10 (group 1) and eight patients had a lower limb HJHS ≥ 10 (group 2). One patient had bilateral total knee arthroplasty and another one had a unilateral total knee arthroplasty, both in group 2, because of the knee haemophilic arthropathies. No difference was found between the PWMH of group 1 and the PWMH of group 2, with regard to age (*p* = 0.67), weight (*p* = 0.52), and height (*p* = 0.79).

### 3.2. Gait Score Analysis

Table 2 presents the comparison of the gait scores (GPS and GVS) between the controls, group 1, and group 2. The GPSs were not different between the three groups. Both knee and ankle flexion/extension GVSs were increased in group 2 compared to the controls (*p* = 0.02 and *p* = 0.04, respectively).

### 3.3. Spatial/Temporal Assessment

The spatio-temporal parameters of all of the groups are presented in Table 3. The PWMH in group 2 had a significant increase in their stance phase, double support duration, and stride width compared to the control group and group 1 (*p* ≤ 0.01).

### 3.4. Correlation between Gait Scores and Clinical/Radiological Parameters

Table 4 presents the correlation matrix between the scores. In the PWMH, no correlation was found between the GPS and lower limb HJHS. Very low correlations were found for ankle GVS with ankle Pettersson sub-score (r^2^ = 0.250; *p* = 0.004), and ankle HJHS sub-score (r^2^ = 0.150; *p* = 0.04). For the knee, a very low correlation was also found between the knee GVS and its HJHS sub-score (r^2^ = 0.290; *p* < 0.0001).

## 4. Discussion

Despite an assumed lower risk of bleeding, some PWMH tended to develop authentic haemophilic arthropathies [10,33]. However, specific assessments of PWMH are lacking. To our knowledge, the present study is the first to focus on 3DGA in adult PWMH. The main results were that PWMH with the highest HJHS score (group 2) presented significant gait deviations concerning the knee and ankle gait scores compared to the healthy controls. They also exhibited spatial/temporal alteration compared to both the healthy controls but also compared to the PWMH < 10 (group 1). However, no gait alteration was found in group 1 compared to the healthy controls.

One-third of our patients had a poor joint status (mean HJHS: 22.3 ± 11.3) mainly due to ankle arthropathy (mean ankle Pettersson score: 7.5 ± 3.6), regardless of age, level of clotting factors, or type of haemophilia. This is consistent with previous studies showing that some PWMH presented haemophilic arthropathy [5,8,9,11]. For example, Måseide et al. recently reported in a cohort of 145 PWMH that 25% had a poor joint status [11].

The GPS quantified the overall impact of moderate haemophilia on walking in comparison to a control group. The GPS is a widely used index and has previously been applied in the context of haemophilia assessed with 3D gait analysis [18,21,24,25]. We found no difference regarding the GPS for both groups of PWMH. These findings are consistent with Putz et al. [18] who calculated the GPS in a group of 18 patients with haemophilia (mixture of severe and moderate haemophilia, in unknown proportion) with a higher rate of joint impairment (mean overall HJHS in their study of 18.8 ± 12.0 vs. 9.3 ± 11.6 in ours). Putz et al. computed another global gait score (i.e., the Gait Deviation Index (GDI) [18,24]), which did not reveal significant differences compared to the controls. Previously, Forneris et al. [21] computed both the aforementioned gait scores on a sample of six children with moderate haemophilia and found no difference compared to the controls [21]. However, we found that for the GVS of sagittal ankle and knee kinematics and gait alterations when considering the patients with the most significant joint damage (group 2). Consistently, Lobet et al. found, in a group of 31 adults with multiple joint impairments, a significant alteration in the active ankle range of motion and a trend in the knee range of motion [34]. In contrast, Putz et al. found no alteration in the lower-limb active range of motion in their study [18]. These conflicting results may be due to the heterogeneity of the included patients and due to the poor comparability between studies. Another explanation may also be due to the different treatments of the patients. Indeed, all of our patients had on-demand therapy whereas in the study by Putz et al. [18], all of the patients had prophylaxis, and in that by Forneris et al. [21], 50% had prophylaxis, 40.5% had on-demand therapy, and 9.5% had immune tolerance induction.

Regarding the spatiotemporal parameters, we found that the PWMH in group 2 had a significant increase in their stance phase, double support duration, and stride width compared to the control group and the group 1 (*p* < 0.01). Interestingly, a previous study of Lobet et al. [35] also showed an increase in the stance phase duration in a group of 10 patients with ankle arthropathies. They did not provide the radiographic parameters or the clinical severity of the arthropathies, which prevented us from comparing our findings. However, despite a higher HJHS, Putz al. found no modification of the stance phase [18]. They only reported a decrease in the walking velocity and the step length, whereas we only reported a decreased trend of those parameters in our study (*p* = 0.06).

We found an absence of correlation between HJHS and GPS (*p* = 0.06), which is in contradiction with Putz et al., who reported a high correlation (r = 0.63; *p* = 0.005) [18]. This discrepancy could be attributed to their higher proportion of patients affected by a severe phenotype of haemophilia. However, Putz et al. also reported an absence of correlation between the HJHS and the GDI (*p* = 0.11) [18]. Both the GDI and GPS are scores aiming to quantify the overall kinematic deviation from normal values. As a linear relationship between both scores has previously been demonstrated [18,24], the contradicting results of the study by Putz et al. may not be interpreted as a relationship between clinical severity and gait impairment. However, we found a very low but significant correlation between the knee and ankle HJHS sub-scores and their respective GVSs, which seems to be a significant weak link between the joint structure and function. We have also reported a very low but significant correlation between the ankle GVS and the ankle Pettersson score (r^2^ = 0.250; *p* = 0.004), which might suggest a poor link between function and radiography. No such relation was reported by Lobet et al. for patients with severe haemophilia [36].

Finally, our study suffered from several limitations. Our sample size was relatively small and thus reduced its statistical power. The inclusion of more patients is challenging since PWMH represents the smallest proportion of patients with haemophilia. Further investigations should be performed on a multicentric level. Furthermore, interpretability remains difficult due to the heterogeneity of the joint deterioration and the inter-individual heterogeneity of the walking patterns. Moreover, GPS and GVS are global scores over the whole gait cycle, which does not exclude that some outcomes may have varied specifically. The choice of the Pettersson score could also be arguable and MRI imaging could be an interesting choice due to its accuracy to detect early joint damage [37]. However, Brunel et al. mitigated the use of MRI since they reported a low correlation between the medical images and ankle kinematic parameters [38]. Ultrasound might be a worthy alternative as it has highlighted a high correlation between HJHS in PWMH, which is helpful to the early detection of haemophilic arthropathies [11,39]. Nevertheless, X-ray imaging remains one of the main parameters of the follow-up of patients with haemophilia because of its accessibility, low cost, and ease of interpretation [31]. Furthermore, the inclusion of patients over 60 is another limitation. Over 60 years of age, the risk of the arthropathy of other natures increases [40]. Nevertheless, group 1 and group 2 did not differ according to age, and the X-rays were analysed independently by two experienced physicians to confirm the haemophilic origin of the arthropathy.

## 5. Conclusions

Our study showed that patients with moderate haemophilia presented significant gait deviations in the case of poor lower limb joint status (i.e., lower limb HJHS ≥10). Further investigations in a larger group of PWMH should be performed to confirm these findings and precise detail the heterogeneity of PWMH regarding their lower limb impairments and function. These results underline the importance of 3D gait analysis for early diagnosis and monitoring haemophilic arthropathies. It also may encourage for prophylactic treatments for PWHM with the bleeding phenotype.

## Figures and Tables

**Table 1:** The characteristics of the studied population and comparison between patients with a lower limb HJHS < 10 (group 1) and a lower limb HJHS ≥ 10 (group 2) (*t*-tests for independent variables or Mann–Whitney tests).

	Patients with Moderate Haemophilia (*n* = 24)	Group 1 (*n* = 16)	Group 2 (*n* = 8)	*p*
Haemophilia A/B (*n*)	15/9	8/8	7/1	0.08 ^a^
Level of clotting factor, % (mean ± SD) (min–max)	3.0 ± 1.0 (1.0–5.0)	3.1 ± 0.9 (2.0–5.0)	2.8 ± 1.3 (1.0–4.5)	0.57
Age, years (mean ± SD) (min–max)	44.3 ± 16.1 (20–72)	42.8 ± 16.2 (20–72)	47.4 ± 16.7 (25–65)	0.67
Weight, kg (mean ± SD) (min–max)	75.9 ± 15.0 (48.0–112.0)	73.8 ± 15.5 (48.0–112.0)	75.7 ± 7.7 (65.0–85.0)	0.52
Height, cm (mean ± SD) (min–max)	173.0 ± 6.6 (160.0–185.0)	173.3 ± 6.5 (164.0–185.0)	172.6 ± 7.3 (160.0–182)	0.95
Body mass index, kg/m^2^ (mean ± SD) (min–max)	25.30 ± 4.9 (18.0–38.8)	24.6 ± 5.2 (18.0–38.8)	26.8 ± 3.9 (22.0–35.1)	0.17
Total HJHS (mean ± SD) (min–max)	10.4 ± 14.0 (0–56)	2.9 ± 3.5 (0–9)	22.3 ± 11.3 (11–41)	<0.0001
Lower limb HJHS (mean ± SD) (min–max)	9.0 ± 11.1 (0–41)	2.7 ± 3.4 (0–9)	18.5 ± 6.8 (10–41)	<0.0001
Knee HJHS sub-score (mean ± SD) (min–max)	3.0 ± 4.2 (0–16)	1.4 ± 1.8 (0–6)	6.1 ± 5.8 (0–16)	0.03
Ankle HJHS sub-score (mean ± SD) (min–max)	4.4 ± 6.7 (0–22)	0.7 ± 1.4 (0–4)	11.8 ± 7.0 (2–22)	<0.0001
Pettersson knee sub-score (mean ± SD) (min–max)	2.0 ± 2.3 (0–7.5)	1.7 ± 2.0 (0–7.5)	2.6 ±2.8 (0–7.0)	0.61
Pettersson ankle sub-score (mean ± SD) (min–max)	3.3 ± 3.8 (0–12)	1.3 ± 1.5 (0–5.5)	7.5 ± 3.6 (2.5–12)	<0.0001

^a^ Kruskall–Wallis test for the qualitative variable.

**Table 2:** The comparison of the gait scores between the controls and patients with moderate haemophilia lower limb HJHS < 10 (group 1) and lower limb HJHS ≥ 10 (group 2). The ANOVA test followed by the Bonferroni post hoc test.

	Group 1 (*n* = 16)	Group 2 (*n* = 8)	Control Group from the Normative Dataset (*n* = 30)	*p*
Gait Profile Score	4.73 ± 0.32	5.34 ± 0.41	4.21 ± 0.41	0.20
Knee Gait Variable Score (degrees)				
- Flexion/Extension	5.63 ± 0.46	7.25 ± 0.69 ^a^*	4.56 ± 0.63 ^a^*	0.02
Ankle Gait Variable Score (degrees)				
- Flexion/Extension	4.56 ± 0.45	5.97 ± 0.64 ^a^*	3.74 ± 0.57 ^a^*	0.04

Bonferroni post hoc test: ^a^ Comparison between group 2 and the controls; * *p* ≤ 0.05.

**Table 3:** The comparison of the spatial/temporal parameters between the controls, patients with moderate haemophilia with lower limb HJHS < 10 (group 1), and lower limb HJHS ≥ 10 (group 2). The ANOVA test followed by the Bonferroni post hoc test.

	Group 1 (*n* = 16)	Group 2 (*n* = 8)	Control Group from the Normative Dataset (*n* = 30)	*p*
Walking velocity (m/s)	1.24 ± 0.06	1.20 ± 0.08	1.28 ± 0.07	0.06
Cadence (steps/min)	56.8 ± 1.47	52.1 ± 2.08	59.0 ± 1.86	0.06
Stance phase (%gait cycle)	60.6 ± 0.47 ^a^**	62.8 ± 0.65 ^a^**^, b^**	59.6 ± 0.59 ^b^**	0.004
Double support (ms)	10.2 ± 0.50 ^a^**	12.60 ± 0.69 ^a^**^, b^**	9.54 ± 0.63 ^b^**	0.006
Stride Width (m)	0.12 ± 0.01 ^a^**	0.15 ± 0.01 ^a^**^, b^**	0.11 ± 0.01 ^b^**	0.002
Step length (m)	0.66 ± 0.02	0.59 ± 0.03	0.66 ± 0.03	0.18

Bonferroni post hoc test: ^a^ Comparison between group 1 and group 2; ^b^ Comparison between group 2 and the controls; ** *p* ≤ 0.01.

**Table 4:** The Spearman correlation of the global scores and joint sub-scores in patients with moderate haemophilia (*n* = 24).

	Lower Limb HJHS	KHJHS	KPS	AHJHS	APS
GPS	r^2^ = 0.100 *p* = 0.06				
Knee GVS		r^2^ = 0.290 * p < 0.0001	r^2^ = 0.01 *p* = 0.61		
Ankle GVS				r^2^ = 0.150 * p = 0.04	r^2^ = 0.250 * p = 0.004

Abbreviations: HJHS: Haemophilia Joint Health Score; KHJHS: Knee HJHS; AHJHS: Ankle HJHS; KPS: Knee Pettersson Score, APS: Ankle Pettersson Score; GPS: Gait Profile Score; GVS: Gait Variable Score. * Indicates a significant difference inferior to the threshold 0.05.

## Data Availability

The data presented in this study are available on request from the corresponding author. The data are not publicly available due to ethical reasons.
